# An Operation for Pyonephrosis

**Published:** 1883-03

**Authors:** 


### Original Translations and Abstracts.

### AN OPERATION FOR PYONEPHROSIS.

Operative interference in surgical diseases of the kidney has been attended, since the time of G. Simon, with such favorable results, technically speaking, that the indications for such interference have been widely extended; so widely, indeed, that the *healthy kidney* has been extirpated under certain circumstances.

[For a case in point see the January number of THE INDEPENDENT PRACTITIONER, page 12. Ed. L. H. H.]

On the 31st October, 1882, I was called by Dr. Maltzky to see a woman, æt. 34, confined to her bed by intense pain which made movement impossible.

The patient stated that, up to four weeks previous, she had been well, except "that she had felt something" in the left lumbar region that had given her slight discomfort since early youth. She also noticed that she was forced to micturate very frequently. The urine was always cloudy, and stunk after standing a short time.

Menstruation regular; twice married; sterile.

Four weeks previous, violent pains commenced in the left side, accompanied by marked febrile symptoms, so that she was forced to take to her bed. The temperature underwent morning remissions and evening exacerbations up to 39 degrees (102.°1 Fahr.) Patient well nourished and robust.

The results of an examination under anæsthetics were as follows: the left side of the abdomen was markedly distended by a tumor, somewhat ovoid in form, the long axis running from above and outwards, downwards and inwards. Vertically, it filled all the left side between the margin of the ribs and the iliac crest. At its lower border it reached the symphysis pubis. The internal border of the tumor extended two inches to the right of the mesial line. The mass was exceedingly elastic, fluctuating only at its centre. Concerning mobility, nothing positive could be ascertained either when the patient was supine or when she lay upon the right side. The percussion note over the tumor was dull; but along the edge of the ribs and near the pelvic brim it was bounded by a zone of tympanitic resonance. In the lumbar region it was dull-tympanitic. The lower, elastic, fluctuating portion of the tumor could be felt through the vagina. We could make out no connection between the genital organs and the tumor. The uterus was antiflexed and freely movable. The urine for twenty-four hours amounted to 1800-1900; cloudy, ammoniacal, the purulent sediment showing, macroscopically, small, whitish granules.

A *microscopical examination* revealed a *débris* of large epithelia, apparently from the pelvis of the kidney, commingled with an amorphous detritus wherein were recognized multitudes of bacteria and a few pus corpuscles. The macroscopic whitish granules consisted of a conglomerate of epithelia and detritus, incrusted with calcic phosphate. The filtered urine contained large quantities of albumin.

There was nothing abnormal in the thoracic viscera. The absence of cardiac hypertrophy or dilatation is to be especially noticed.

From these data, there was no doubt but what we had to deal with an enormous pyonephrosis and that the removal of such a condition was necessary to the life of the patient. From the history we learned that disease of the left kidney had existed from youth; from urinary analysis we concluded that, in all probability, since products of this diseased process still reached the bladder, there could be no question of any complete closing of the ureter. For, had there been no communication between the left kidney and the bladder, then all the morbid urinary ingredients must have come from the right kidney. This would mean an intense pyelitis on that side, and of this we could not find the slightest evidence in our patient.

I concluded not to extirpate, but to make a fistulous opening from the pelvis of the kidney through the abdominal walls.

Operated on November 2nd.—Abdominal incision 10 ctm. long, following a line from angle of the eleventh rib to the centre of a line joining the left iliac spine and navel.

This cut opened the peritoneal cavity. I should have liked to carry the incision further back toward the axillary region so as to incise the tumor from the extra-peritoneal side, but I was forced to carry the cut thus far forward, since fluctuation was distinct at this point alone.

Upon opening the abdominal cavity, we saw, at the inner margin of the wound, the colon descendens which passed down over the anterior wall of the tumor.

The boundary of the parietal peritoneum upon the anterior surface of the mass was 4 ctm. beyond the outer lip of the incision. After pushing the descending colon in toward the median line, and underneath the inner border of the wound, I put in two strong sutures through the latter and the tumor, whose tension kept the peritoneal cavity closed above and below.

Then I thrust a long, curved trocar into the kidney, from which immediately flowed a large quantity of stinking purulent fluid.

After the flow ceased I dilated the opening on both sides (about 3 ctm. each), parallel with the abdominal incision, thoroughly washed out the renal sac with thymol water, carefully cleaned the peritoneum, and, finally, sewed the edges of the cyst's wound to the lips of the abdominal incision, the sutures embracing the parietal peritoneum.

The tumor was freely movable, but did not collapse as I expected; and despite repeated washing out, till the thymol water flowed away perfectly clear, a thin stream of purulent fluid still continued to ooze from the lower part of the incised cyst. This led me to the supposition that I had incised *not* the pelvis of the kidney, but an enormously extended cyst of a calyx. This supposition became a certainty when, on inserting my finger, *not such a calyx as would have existed were there a dilated pelvis*, but only an opening the size of a lead pencil was discovered. The latter opening afforded a communication between the pelvis of the kidney and the cyst I had just opened.

The operation concluded with the insertion of two drainage tubes, the wound being dressed with iodoform. The subsequent condition of the patient was encouraging. No trace of fever or sign of peritonitis showed themselves. The temperature on the evening of the day of operation was 35 9-10 degrees C. The urine, drawn by the catheter, was first clear and acid in reaction; but toward the close of the act pus flowed with the same stinking odor of that drawn off at the operation. This proved that there was a free communication between the bladder and the affected kidney. Next morning the temperature was 36 9-10 degrees; the pulse 84; the patient comfortable after several hours of quiet sleep; urine acid, and almost clear. That evening temperature 35 degrees; pulse 84; 1,200 cubic cent. of urine for twenty-four hours. Gave a hypodermatic injection of morphia that same evening, since the patient had a desire to go to stool. Next morning (Nov. 4th) I found the patient greatly altered for the worse. Though the temperature had remained at 36 9-10 degrees, the pulse had risen from 84 to 128, and the respirations were increased to 36. There was slight somnolence. The face was pale and like that of collapse ; now and then slight convulsive movements in the fingers occurred. At most the amount of urine was 200 c.c. The body was anæsthetic ; there was no vomiting. In the evening the patient was comatose ; pulse scarcely perceptible ; hurried breathing and suppression of urine were followed by death. As a cause of the fatal issue peritonitis had to be excluded ; while uræmia was clearly evidenced. The autopsy revealed the following : lungs pale and extensively œdematous ; heart small from imperfect development of the walls and papillary muscles. Maximum thickness of the wall of the left venticle 11 mm ; color brownish. On opening the abdomen a *perfectly normal* peritoneum was found. The descending colon, running directly downward from the free margin of the ribs, was found pushed far forward and toward the mesial line by a tumor in the retro-peritoneal tissue in the region of the psoas. The colon adhered to the median lip of the wound made at the operation.

The *left kidney* was transformed into an immense tumor, which had developed between the layers of the meso-colon, which it had separated from each other in its growth. With increase of the tumor, the outer layer of the meso-colon was pushed so far forward that it was a finger's breadth to the side of the outer edge of the wound. This line also formed the lateral boundary of the peritoneal covering of the tumor. The capsule was, along with the neighboring connective and adipose tissue, converted into a thick, tough, cicatricial mass. After dividing this tissue we endeavored to loosen the capsule from the kidney, which could not be done without tearing the renal parenchyma, so adherent was it in spots. The kidney, loosed from the adherent capsule, consisted of a flaccid, very thin walled sac, 18 c. in length and 7 c. in breadth. The remainder of the parenchyma was friable, yellowish in color, and at points had undergone fatty degeneraton. The ureter was normal in calibre—showed no obstruction from pelvis to bladder. On section of the kidney there flowed a considerable quantity of purulent fluid. The pelvis of the organ, into which an opening was made at the operation, was somewhat dilated, but the calyx, which freely communicated with the pelvis, was transformed into an immense cavity whose walls were intact. Thus the kidney formed a kind of multilocular cyst. Now, I saw that my incision did not open the pelvis of the kidney, but the cystic calyx, which latter communicated with the pelvis by an opening the size of a cherry pit. The insertion of the ureter was slit-like and lay in the anterior wall. When the ureter was cut longitudinally, its posterior wall appeared as a prominence, as a valve, in the lumen of the pelvis. The *right kidney* was very much diminished in size and showed well marked granular atrophy; size 8x3½ cent. Its pyramidal portion was especially decreased from hydronephrotic distension of the pelvis of the kidney. The ureter was perfectly normal in its course and in its place of insertion. It measured 1½ cent. in diameter. The bladder was normal in size; the mucous membrane showed only a few pigment spots. Three intramural fibroids the size of a cherry were found in the uterus. The remains of an old peritonitis were found in adhesions between the left ovary and the sigmoid flexure. None of these lesions interfered in any way with the ureters. The liver was small and very flabby; the parenchyma exceedingly clouded as if cooked. The spleen was small, flaccid, atrophied, and its capsule wrinkled.

[Concluded in April Number.]

